# Effect of glucagon-like peptide-1 receptor agonists on major adverse cardiovascular events in patients with inflammatory bowel disease

**DOI:** 10.1093/crocol/otaf056

**Published:** 2025-08-12

**Authors:** Michael Saadeh, Apoorva Krishna Chandar, Sadeer Al-Kindi, Vu Quang Nguyen, Revital Gorodeski Baskin, Jeffry Katz, Fabio Cominelli, Miguel Regueiro, Emad Mansoor

**Affiliations:** Internal Medicine, Case Western Reserve University/University Hospitals Cleveland Medical Center, Cleveland, OH, United States; Digestive Health Institute, University Hospitals Cleveland Medical Center, Cleveland, OH, United States; Department of Gastroenterology, Case Western Reserve University, Cleveland, OH, United States; DeBakey Heart and Vascular Center, Houston Methodist, Houston, TX, United States; Digestive Health Institute, University Hospitals Cleveland Medical Center, Cleveland, OH, United States; Department of Gastroenterology, Case Western Reserve University, Cleveland, OH, United States; Division of Endocrinology Center for Diabetes and Obesity, University Hospitals Cleveland Medical Center, Cleveland, OH, United States; Digestive Health Institute, University Hospitals Cleveland Medical Center, Cleveland, OH, United States; Department of Gastroenterology, Case Western Reserve University, Cleveland, OH, United States; Digestive Health Institute, University Hospitals Cleveland Medical Center, Cleveland, OH, United States; Department of Gastroenterology, Case Western Reserve University, Cleveland, OH, United States; Department of Gastroenterology, Hepatology and Nutrition, Cleveland Clinic, Cleveland, OH, United States; Digestive Health Institute, University Hospitals Cleveland Medical Center, Cleveland, OH, United States; Department of Gastroenterology, Case Western Reserve University, Cleveland, OH, United States

## Introduction

Inflammatory bowel disease (IBD), which is comprised of ulcerative colitis (UC) and Crohn’s disease (CD), is a chronic immune mediated condition characterized by relapsing and remitting intestinal inflammation. IBD is associated with several extraintestinal manifestations and comorbid conditions. It has been demonstrated that patients with IBD are at an increased risk of cardiovascular disease such as myocardial infarction (MI), cerebrovascular events, and heart failure, collectively known as major adverse cardiovascular events (MACE).^1^^,^^2^^,^^3^ Recent studies have shown that glucagon-like peptide-1 receptor agonists (GLP-1RA), an anti-diabetes and anti-obesity drug class, have a beneficial impact on cardiovascular outcomes in patients with metabolic disorders such as obesity and type II diabetes mellitus.^4^^,^^5^ We hypothesized GLP-1RA will reduce MACE in patients with IBD.

## Methods

We utilized the US Collaborative Network within the TriNetX database to create our cohorts from 67 HCOs and over 110 million patients. Patients were identified in the TriNetX database using International Classification of Disease, 10^th^ Revision (ICD-10) codes, and medications were identified using normalized names for medical prescriptions (RxNorm). Patients with IBD were defined as individuals aged 18 years or older carrying at least two instances of UC or CD diagnoses as well as ever having exposure to at least one IBD-related medication and were categorized into 2 subgroups based on their exposure to GLP-1RA. Cases were IBD patients on a GLP-1RA, while controls were IBD patients without exposure to GLP-1RA. The two cohorts were propensity score matched for demographics and comorbidities, including traditional risk factors for atherosclerotic disease such as age, sex, race, body mass index, type 2 diabetes mellitus, nicotine dependence, cardiovascular and pulmonary diseases, chronic kidney disease, and dyslipidemia, in addition to cardioprotective medications and specific IBD medications. The outcome of interest was MACE, defined as a composite of cerebrovascular accident, acute heart failure exacerbation, and acute MI 6 months to 5 years after the index event of GLP-1RA use. Secondary outcomes were the individual components of the composite endpoint. We stratified our analysis by the inclusion or exclusion of prior MACE. Chi-square and t-tests were used for significance testing.

## Results

Excluding prior MACE, there were 12,917 cases and 255,110 controls. After propensity matching, there were 10,055 cases and controls who were evenly matched (Table 1). There were similar numbers of cases and controls before and after matching in the inclusion of prior MACE group. When compared to patients with IBD and no exposure to GLP-1RA, those with IBD on GLP-1RA were less likely to have new onset MACE (OR 0.50, 95% CI 0.44-0.57). Examining secondary outcomes, those with IBD on GLP-1RA were less likely to have the individual components of MACE, specifically MI (OR 0.51, 95% CI 0.42-0.62), acute heart failure exacerbation (OR 0.41, 95% CI 0.34-0.50), and CVA (OR 0.52, 95% CI 0.44-0.62). Furthermore, this beneficial effect persisted even when including patients with prior MACE (OR 0.55, 95% CI 0.50-0.60), specifically MI (OR 0.56, 95% CI 0.49-0.65), acute heart failure exacerbation (OR 0.45, 95% CI 0.39-0.53), and CVA (OR 0.58, 95% CI 0.51-0.66; Figure 1).

## Discussion

In this retrospective cohort study using a large healthcare database comprising over 110 million patients, we found that the use of GLP-1RA was associated with decreased MACE.

While patients with IBD are known to have an increased risk of MACE, the exact pathophysiology of this association is not fully known. In both atherosclerosis and IBD, chronic inflammation-mediated endothelial dysfunction is likely contributory to cardiovascular disease (CVD). In combination with genetic predispositions and dysbiosis, chronic inflammation leads to dysregulation of inflammatory cytokines such as tumor necrosis factor alpha (TNFα), interleukin-1 (IL-1), and vascular epithelial growth factor, resulting in damage of the vasculature endothelium and accelerated atherosclerosis.^6^ Furthermore, studies have identified an association between surrogates for disease activity such as inflammatory markers, IBD specific hospitalizations, and steroid use, and incidence of ASCVD.^7^

While the pathophysiology suggests an atherosclerotic mediated pathway of MACE in patients with IBD, current ASCVD risk assessment models do not accurately estimate risk in the IBD population.^8^ This is potentially secondary to the use of traditional risk factors, such as hypertension, diabetes, and dyslipidemia, which have been found at lower rates in patients with IBD despite increased risk of MI.^3^ Nontraditional risk factors such as surrogates for disease activity may more accurately suggest risk of MACE in patients with IBD.

Without reliable risk assessment models, therapies which may reduce the risk of MACE are even more important. One such example is GLP-1RA, which have been shown to promote anti-inflammatory states via macrophages and increasing IL-10, an anti-inflammatory cytokine, as well as decreasing TNFα and IL-1β.^9^ The incretin hormones, including GLP-1 and glucose-dependent insulinotropic peptide, have been shown to decrease activation of NF-κB, leading to decreased pro-inflammatory cytokines including IL-1β, IL-6, and TNFα. GLP-1RA are associated with significant weight loss and reduction of insulin resistance, as GLP-1 increases expression of glucose transporters and modulation of lipid metabolism, and in conjunction with its anti-inflammatory activity, leads to improvement in insulin sensitivity.^10^

Limitations of our study included the use of billing codes, retrospective cohort analysis, and inability to detect MACE and MACE management if patients received care outside of HCOs covered by TriNetX. Notable strengths including a large, US-based sample size and rigorous propensity score matching.

In conclusion, our study suggests GLP-1RA are associated with decreased risk of MACE in patients with IBD, a high-risk population. Further research is needed to investigate the impact of extraintestinal inflammation in patients with IBD, as well as the role of GLP-1RA in modulating inflammation in IBD patients with relation to cardiometabolic outcomes.

## Data Availability

The de-identified electronic health-record data used in this analysis were obtained under license from the TriNetX Research Network (Cambridge, MA, USA). Because of contractual and privacy restrictions, the raw data are not publicly available. Qualified researchers may obtain access to comparable data sets by contacting TriNetX (https://trinetx.com) and entering into a data-use agreement. Details the demographics, comorbidities, and medications after matching the cases and controls. Abbreviations: IBD, inflammatory bowel disease; GLP-1RA, patients on glucagon-like peptide-1 receptor agonists; TNFα, tumor necrosis factor alpha. The risk of major adverse cardiovascular events (MACE) in inflammatory bowel disease (IBD) patients on glucagon-like peptide-1 receptor agonists (GLP-1RA) compared to IBD patients without exposure to GLP-1RA. The beneficial impact persists even when including patients with prior MACE.
